# Efficacy and safety of *Ojeok-san* plus *Saengmaek-san* for gastroesophageal reflux-induced chronic cough: protocol for a pilot, randomized, double-blind, placebo-controlled trial

**DOI:** 10.1186/s13063-019-4030-z

**Published:** 2020-01-29

**Authors:** Yeon Hee Bhang, Kwan-Il Kim, Jaehyo Kim, Junmo Ahn, Hwan-Su Jung, Changsop Yang, Seok-Jae Ko, Youngmin Bu, Jae-Woo Park, Kyoung Sun Park, Hee-Jae Jung, Jun-Hwan Lee, Beom-Joon Lee

**Affiliations:** 1 Division of Allergy, Immune and Respiratory System, Department of Internal Medicine, College of Korean Medicine, Kyung Hee University, 26 Kyung Heedae-ro, Dongdaemun-gu, Seoul, 02447 Republic of Korea; 2 Department of Clinical Korean Medicine, Graduate School, Kyung Hee University, 26 Kyung Heedae-ro, Dongdaemun-gu, Seoul, 02447 Republic of Korea; 3 College of Korean Medicine, Sang-Ji University, 83 sangjidae-gil, Wonju-si, Ganwondo 26339 Republic of Korea; 4 Clinical Medicine Division, Korea Institute of Oriental Medicine, Daejeon, 34054 Republic of Korea; 5 College of Korean Medicine, Kyung Hee University, 26 Kyung Heedae-ro, Dongdaemun-gu, Seoul, 02447 Republic of Korea; 6 Korean Medicine Life Science, University of Science & Technology (UST), Campus of Korea Institute of Oriental Medicine, 1672 Yuseong-daero, Yuseong-gu, Daejeon, 34054 Republic of Korea

**Keywords:** Chronic cough, Gastroesophageal reflux disease (GERD), Herbal medicine, *Ojeok-san*, *Saengmaek-san*, Randomized controlled trial

## Abstract

**Background:**

Gastroesophageal reflux disease (GERD) is a major cause of chronic cough. GERD-induced chronic cough is difficult to diagnose because some patients do not complain of any gastrointestinal (GI) reflux symptoms. Although chronic cough due to GERD is highly prevalent, no effective treatment is currently available, especially for GERD-related cough without GI symptoms. Because the herbal medicines *Ojeok-san* and *Saengmaek-san* can effectively treat GERD and cough, we aim to evaluate the efficacy and safety of a combination of these components for relieving chronic cough due to GERD.

**Methods/design:**

This is a study protocol of a randomized, double-blind, placebo-controlled, single-center pilot trial. After a 1-week run-in period, a total of 30 patients with GERD-induced chronic cough will be randomly allocated to an intervention group (*n* = 15) or a placebo group (*n* = 15). Participants will receive 5.76 g of *Ojeok-san* plus *Saengmaek-san* or a placebo three times per day for 6 weeks. The primary outcome measures, which are the frequency and severity of cough, will be recorded using a cough diary. The secondary outcome measures will include a cough visual analogue scale, the Leicester Cough Questionnaire (Korean version), the Gastrointestinal Symptom Rating Scale, the Hull Airway Reflux (hypersensitivity) Questionnaire, the Pattern Identification for Chronic Cough Questionnaire, the Pattern Identification for Gastroesophageal Reflux Disease, and safety testing*.* Adverse events will also be reported.

**Discussion:**

This will be the first clinical trial to explore the use of herbal medicines for GERD-related chronic cough, including patients without GI reflux symptoms. This study will provide useful evidence regarding the efficacy and safety of *Ojeok-san* plus *Saengmaek-san* treatment. In addition, this trial will offer a scientific basis for the combination of herbal medicines. This study will also provide important data for conducting a larger-scale clinical trial on GERD-induced chronic cough.

**Trial registration:**

This trial has been registered with Clinical Research Information Service (CRIS) of South Korea (http://cris.nih.go.kr; registration number KCT0003115). Registered August 28, 2018.

## Background

Cough is the most common respiratory symptom encountered in an outpatient practice. This symptom can be classified by duration as follows: acute, < 3 weeks; subacute, 3–8 weeks; and chronic, ≥ 8 weeks [1]. Of these, chronic cough is of interest to respiratory medicine clinicians because it is associated with a poorer quality of life and complications such as nausea, chest pain, rib fractures, urinary incontinence, syncope, and depression [2].

The main causes of chronic cough include upper airway cough syndrome (UACS), cough variant asthma (CVA), and gastroesophageal reflux disease (GERD). GERD is the second or third most common cause of this condition [3, 4], although some reports present GERD as the most common cause, occurring in 30–40% of patients [5, 6]. The mechanism by which GERD induces cough involves vagal mediation of the esophageal-tracheal-bronchial reflex triggered by acid reflux into the lower esophagus and aspiration [7, 8]. The incidence of chronic cough due to GERD ranges from 5% to 41% among adults [9, 10].

The prevalence is varied because of the differences between populations. Moreover, GERD-related cough is often difficult to diagnose because it has no symptoms that are caused by reflux. It has been reported that approximately 70% of patients with reflux-related chronic cough are without gastrointestinal (GI) reflux symptoms such as heartburn [10, 11].

Patients with GERD-related cough who do not complain of GI symptoms show no abnormalities, even with 24-h esophageal pH monitoring, so there is a possibility of misdiagnosis. Therefore, the American College of Chest Physicians (ACCP) guidelines suggested predicting GERD-related cough syndrome by means of excluding other diseases that cause chronic cough [10], and the updated guidelines also support this suggestion [12]. In patients with GERD-related chronic cough without the typical symptoms of GERD, the use of proton pump inhibitors (PPIs) as a standard therapy for GERD might sometimes be ineffective [13]. Therefore, PPIs were recommended against for these patients. The only option available for management of such patients is a few lifestyle changes, such as diet modification or elevating the head of their beds [12]. Therefore, a more effective treatment for GERD-induced chronic cough, with or without GI syndromes such as heartburn or regurgitation, is required.

There are 56 types of insurance-covered Korean medicine (KM) granules used in Korea. Among them, *Ojeok-san* (OJS) and *Saengmaek-san* (SMS) have been used in this study. OJS comprises the most frequently prescribed insurance-covered KM granules, and they are used for digestive disorders. SMS is widely used for relieving cough. This method of prescribing a mixture of herbal medicines is commonly applied in Korea.

OJS is a traditional herbal formula comprising 17 herbal medicines. It is known to treat the symptoms associated with common cold, acute or chronic gastroenteritis, and stomach cramps [14]. OJS has been widely used to treat digestive disorders, including GERD, and is approved by the Ministry of Food and Drug Safety of Korea (MFDS) to treat GERD. According to a recent report, OJS acts on the lungs to improve the symptoms caused by airway inflammation and pulmonary fibrosis [15]. Therefore, we expect that OJS will effectively reduce upper respiratory tract inflammation caused by reflux.

SMS consists of Liriopis Tuber, Ginseng Radix, and Schisandrae Fructus. SMS moisturizes the respiratory mucosa and inhibits coughing, and it has been used mainly for the treatment of dry cough [16]. SMS is also approved by MFDS to treat cough. SMS treats coughs that either are caused by pulmonary fibrosis or are a side effect of radiation [17, 18]. Additionally, recent studies have demonstrated that SMS regulates GI motility by increasing the activity of Cajal cells in the GI tract [19].

Although chronic cough due to GERD is highly prevalent, no effective treatment is currently available, especially for GERD-related cough without GI symptoms. In traditional KM, combination treatment with drugs that are effective for each disease has been widely used for comorbidities such as GERD-induced cough. The effects are clear, but scientific evidence of their therapeutic benefits is lacking. OJS plus SMS as a combination treatment for respiratory and digestive systems has been used in clinics for a long time. In a previous study, we reported cases of GERD-induced chronic cough treated with OJS plus SMS [20]. Moreover, both these drugs are insurance-covered granules; hence, they are economical for patients.

To diagnose patients with reflux-related cough with or without GI symptoms, the present study is recruiting patients in accordance with the guidelines provided by the ACCP. Subsequently, we aim to explore the efficacy and safety of OJS plus SMS for chronic cough due to GERD. Additionally, reflux symptoms will be evaluated to determine if there is a difference in the effectiveness of the drug between the groups with or without the symptoms of reflux.

## Methods/design

**Fig. 1:**
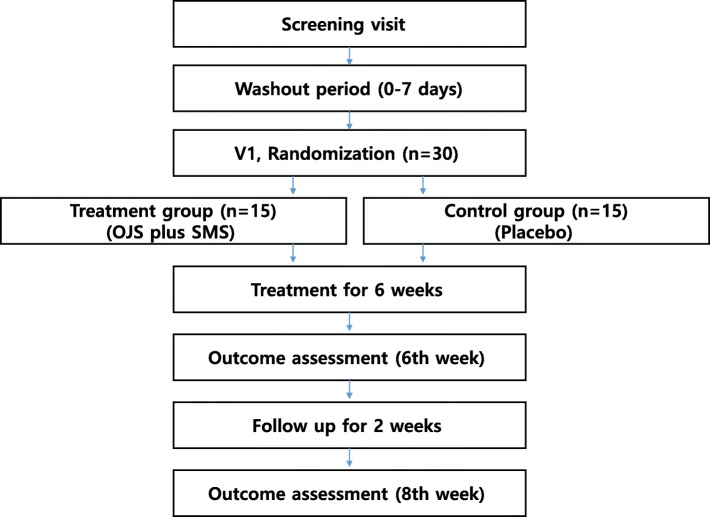
Study flowchart

The present protocol was designed according to the Standard Protocol Items: Recommendations for International Trials 2013 statement (SPIRIT 2013; see Additional file 2). This trial is a randomized, double-blind, placebo-controlled, single-center study that has been authorized by the MFDS (approval number 31617) and registered with the Korean Clinical Trial Registry (KCT0003115). This trial will be conducted at Kyung Hee University Korean Medicine Hospital. A flowchart of the study is shown in Fig. 1.

This trial has been approved by the Institutional Review Board of the Kyung Hee University Korean Medicine Hospital (KOMCIRB 2018-05-017-001). The protocol complies with both the Declaration of Helsinki and good clinical practice guidelines. All eligible patients have to provide signed informed consent prior to enrollment.

### Recruitment

Thirty participants with reflux-induced chronic cough will be recruited through advertisements and referrals at Kyung Hee University Korean Medicine Hospital. The participants deemed eligible after screening with the inclusion and exclusion criteria will be recruited as study subjects and assigned to the experimental or control group in a 1:1 ratio. Each group will be prescribed a drug for 6 weeks.

### Participants

#### Inclusion criteria

We will include participants who (1) are between 19 and 70 years of age, (2) have had a history of cough continuously for > 8 weeks, (3) have been diagnosed with reflux esophagitis within the past year, and (4) have provided written consent to the clinical trial agreement.

#### Exclusion criteria

Participants will be excluded from the study for any of the following reasons:

1. Present with abnormal findings, as established by chest x-ray, pulmonary function test (PFT) with bronchodilator test, fractional exhaled nitric oxide (FeNO), and nasal endoscopy, that might lead to cough
2. Were diagnosed with acute respiratory diseases (including upper respiratory tract disorders) within the past month
3. Were diagnosed with chronic respiratory diseases (e.g., chronic obstructive pulmonary disease, bronchial asthma, bronchiectasis, interstitial lung disease, and other chronic respiratory diseases) within the last 2 years
4. Were diagnosed with Los Angeles classification system grade C or higher GERD within the past year
5. Exhibit symptoms indicative of malignant disease within the GI tract (e.g., severe dysphagia, bleeding, weight loss, anemia, bloody stools)
6. History of surgical or endoscopic antireflux treatment
7. Currently have a disorder such as postnasal drip syndrome, active infection requiring systemic antibiotic therapy, or a blood-clotting disorder
8. Have a lifetime smoking history of ≥20 packs (400 cigarettes)
9. Have used an angiotensin-converting enzyme inhibitor during the previous 4 months
10. Have used cough medicines, glucocorticoids, leukotriene receptor antagonists, anticholinergic drugs, long-acting β_2_-agonists, antihistamines, PPIs, histamine receptor antagonists, mucosa-protective agents, GI motility promoters, antacids, antidepressants, anxiolytics, lower esophageal sphincter agonists, or any herbal medication within the previous 2 weeks
11. Have allergies or sensitivities to the experimental medicine/placebo
12. Have a body mass index < 18.5 kg/m^2^
13. Have an aspartate aminotransferase (AST) or alanine aminotransferase (ALT) level at least twofold higher than the upper limit of normal or a serum creatinine level at least 1.2-fold the upper limit of normal
14. Have a mean cough diary score < 2 during the 1-week run-in period
15. Record < 10 entries in the cough diary during the 1-week run-in period
16. Have a history of malignant tumors (e.g., lung or esophageal cancer) within the last 5 years
17. Are excessive drinkers
18. Are pregnant or breastfeeding
19. Do not consent to use birth control during the trial
20. Have participated in clinical trials for the same disease within the past 3 months
21. Are deemed unsuitable by the investigators

#### Rejection and withdrawal criteria

The rejection and withdrawal criteria will be as follows:

1. Treatment that could affect the clinical trial results without any instruction by the investigator
2. Failure to adhere to the protocol or compliance rate < 80%
3. A serious adverse event (SAE) during the trial
4. Voluntary withdrawal from the trial
5. Use of drugs such as steroids, persistent bronchodilators, and antileukotrienes, anticholinergics, PPIs, histamine receptor antagonists, mucosa-protective agents, GI motility promoters, antacids, antidepressants, anxiolytics, and lower esophageal sphincteric agonist preparations during clinical trials
6. Use of any herbal medications
7. Any other reasons deemed appropriate by the investigators

### Intervention

#### OJS plus SMS

Subjects in the intervention group will be administered a total dose of 5.76 g of OJS (4.35 g/each) plus SMS (1.41 g/each) granules. The participants will be instructed to consume these granules three times per day for 6 weeks. The dosage is based on the requirements of the MFDS. The OJS and SMS granules are manufactured by Han Kook Shin Yak Pharm Co. Ltd. (Nonsan, Chungnam, Republic of Korea), a company that has obtained authorization from the Korea Good Manufacturing Practice. Both the OJS and SMS granules and their ingredients have been approved by the MFDS. These ingredients are presented in Table 1. Voucher specimens will be reserved at the research library of Han Kook Shin Yak Pharm Co. Ltd.

**Table 1:** Composition of *Ojeok-san* and *Saengmaek-san*

*Ojeok-san*	
Ingredients (Latin name)	Amount (g)
Atractylodis Rhizoma	0.95
Ephedrae Herba	0.2
Citri Unshius Pericarpium	0.4
Magnoliae Cortex	0.08
Platycodonis Radix	0.43
Aurantii Fructus Immaturus	0.31
Angelicae Gigantis Radix	0.37
Zingiberis Rhizoma	0.22
Paeoniae Radix	0.27
Poria Sclerotium	0.02
Cnidii Rhizoma	0.3
Angelica dahurica Bentham et Hooker f., Angelica d.	0.31
Pinelliae Tuber	0.22
Cinnamomi Cortex	0.04
Glycyrrhizae Radix et Rhizoma	0.2
Zingiberis Rhizoma Crudus	0.03
Total	4.35
*Saengmaek-san*	
Ingredients (Latin name)	Amount (g)
Liriopis Tuber	0.75
Ginseng Radix	0.30
Schisandrae Fructus	0.36
Total	1.41

#### Placebo

The control group will receive a total of 5.76 g of OJS and SMS placebo granules. The participants will be instructed to consume these granules three times per day for 6 weeks. The placebo is manufactured by the Han Kook Shin Yak Pharm Co. Ltd. in accordance with the placebo guidelines of the MFDS. Although granules do not contain active ingredients, their appearance, taste, and aroma are similar to those of the experimental intervention granules. The OJS and SMS placebos are composed of starch, lactose, citric acid, caramel color, and ginseng flavor powder.

**Fig. 2:**
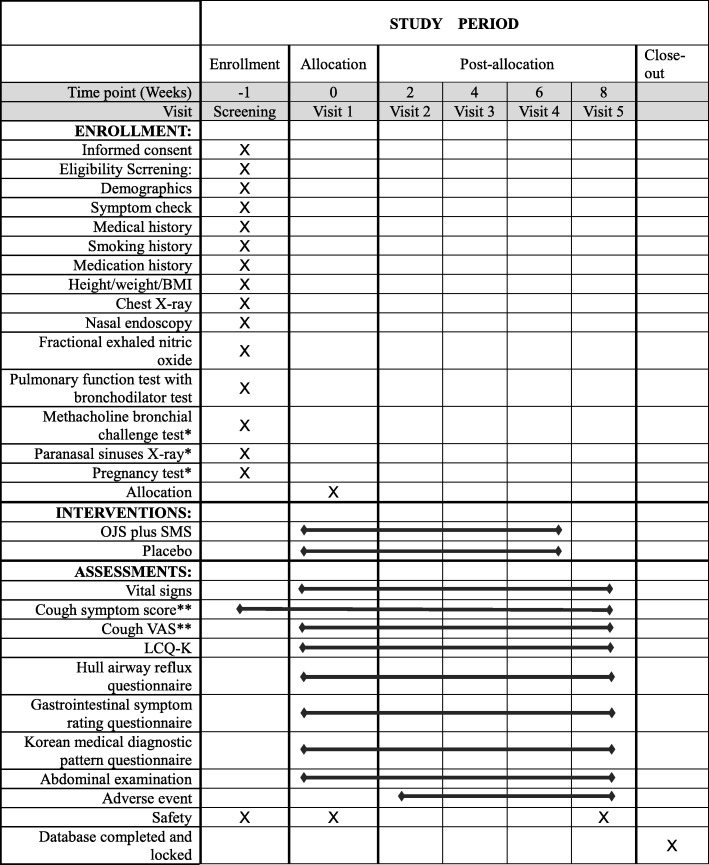
Study process. * The test will be performed as needed, according to the judgment of the investigator. **Cough diary: Two-week self-recording worksheets will be provided at baseline and weeks 2, 4, and 6 after registering the study subjects. The subjects will record data daily and return the worksheets to the investigators on weeks 2, 4, 6, and 8, respectively. BMI, Body Mass Index; LCQ-K, Leicester Cough Questionnaire-Korean version; OJS, Ojeok-san; SMS, Saengmaek-san; VAS, Visual Analogue Scale

All products were packaged by Han Kook Shin Yak Pharm Co. OJS and SMS are packaged in 4.35 g and 1.41 g quantities, respectively, and each placebo is packaged in the same amount. OJS and SMS or OJS placebo and SMS placebo will be provided to each randomized participant at visits 1 (week 0 ± 3 days), 2 (week 2 ± 3 days), and 3 (week 4 ± 3 days). The clinical trial drugs and placebo will be stored at the Korean medical clinical trial center (K-CTC) clinical research pharmacy at Kyung Hee University Korean Medicine Hospital. An independent well-trained pharmacist will be responsible for all the procedures related to drugs. The study process is outlined in Fig. 2.

### Randomization and allocation concealment

Participants will be assigned randomly in a 1:1 ratio to the intervention group (OJS plus SMS group) or the control group (placebo group) using a simple randomization method. An independent statistician will conduct participant randomization using a randomization table created with SAS version 9.1.3 software for Microsoft Windows (SAS Institute Inc., Cary, NC, USA). After receiving an explanation of the study and providing a consent form to participate in the clinical trial, each subject will be given a screening number (S□□-□□□) in order. Subjects who are finally selected to participate in the clinical trial following the screening test and 1-week window period will be given a registration number (R□□ - □□□). A third party will allocate each subject to a group according to the registration number and the random assignment table.

### Blinding

The drug and placebo will be administered by code and by double-blind methods in which the investigator and the participants will not know whether the drug is a test drug or a reference drug. Additionally, the clinical trial pharmacist will be blinded to the treatment allocation. For this purpose, the placebo will be made similar to the experimental medicine with respect to the characteristics, taste, and flavor. The medicine manufacturers directly label the code assigned to the experimental medicine and placebo, and the third party matches the generated random number and code. When a third party registers each study subject, it sends the test number or the control number of the matching test drug to the researcher (e.g., by telephone, text message, or mobile communication application) according to the random number assigned. When a severe or medically significant event occurs, the statistician will uncover the blinding.

### Strategies to improve adherence

To improve participant retention in the study, financial reimbursement will be given to all the participants. Appointments will be scheduled for screening and each visit date. To improve patient compliance, mobile text messages will be sent as a reminder prior to each visit.

### Outcome measures

#### Primary outcome measurement

The primary outcome will be the cough diary score at week 6. The mean cough symptom score in the cough diary during the 1-week run-in period will be set as the baseline score of each participant enrolled and randomized in the trial. We plan to compare the mean scores of the cough diary at week 6 between both groups.

The cough diary is an evaluation scale that divides the severity and frequency of cough into five stages [21]. The subjects will conduct self-evaluations twice daily, and the cough diary will be prepared at 20:00 (daytime, 8:00–20:00) and at 8:00 (nighttime, 20:00–8:00). Patients will be required to evaluate their symptoms twice per day (daytime and nighttime). Cough frequency will be graded on a 5-point scale as follows: 0, no cough; 1, infrequent/occasional; 2, several times; 3, many times; and 4, all the time. Cough severity will be graded on a 5-point scale as follows: 0, symptom is not present.; 1, symptom is present, is not a problem, does not interfere/hinder activity/interactions/sleep in any way; 2, symptom is present, is somewhat of a problem, may interfere/hinder certain activities/interactions/sleep; 3, symptom is present, is a problem, frequently interferes/hinders many activities/interactions/sleep; and 4, symptom is present, is a major concern, very frequently interferes/hinders most activities/interactions/sleep. The total cough score (range, 0–8) is the sum of the daytime and nighttime cough symptom scores.

#### Secondary outcome measurement

##### Cough visual analogue scale (VAS)

The cough VAS is a scale that rates the degree and frequency of coughing on a scale of 0–10 points, with 0 indicating “no cough” and 10 indicating “unbearable cough.” After study registration, the investigator will provide each subject a 2-week self-recording diary at baseline and at weeks 2, 4, and 6. The participants will record daily entries and return the data to the investigators at weeks 2, 4, 6, and 8. We plan to compare the results of mean values of cough VAS evaluated at weeks 2, 4, and 6 between both groups.

##### Leicester Cough Questionnaire–Korean version (LCQ-K)

The LCQ-K is a 19-item questionnaire that is used to measure the quality of life according to cough symptoms [22]. These 19 items are divided into 3 parts: physical, mental, and social. Each item score ranges from 1 to 7 points, and a higher score indicates better health. We will use the validated LCQ-K version [23]. The mean LCQ-K scores between the two groups evaluated at weeks 2, 4, and 6 will be compared.

##### Gastrointestinal Symptom Rating Scale (GSRS)

The GSRS is a widely used and validated self-reported GI symptom scale. It includes 15 symptom items grouped into 5 symptom areas that can be scored on a 7-point scale [24]. The mean GSRS scores between the two groups evaluated at weeks 2, 4, and 6 will be compared.

##### Hull Airway Reflux (hypersensitivity) Questionnaire (HARQ)

The HARQ is a self-reported tool that is used to measure airway hyperresponsiveness due to laryngopharyngeal reflux. Symptoms of airway hypersensitivity caused by laryngeal reflux are grouped into 14 items. Each item is scored on a range of 0–5 points, with a maximum total score of 70 points [25]. We plan to compare the results of mean HARQ scores evaluated at weeks 2, 4, and 6 between both groups.

##### Pattern Identification for Chronic Cough Questionnaire (PICCQ)

The PICCQ is a tool used to identify patterns of chronic cough. Chronic cough is thus classified into four patterns: wind-cold, phlegm-turbidity, fire-heat, and deficiency (lung deficiency and kidney yang deficiency) [26]. Using descriptive analysis, the distribution of participants according to each pattern of differentiation will be depicted as the frequency and ratio.

##### Pattern Identification for GERD

This tool is used to analyze the distribution pattern in patients with GERD who complain of cough as the main symptom. Four GERD patterns have been identified: pattern/syndrome of liver qi invading the stomach, spleen-stomach weakness, spleen-stomach dampness-heat, and stomach yin deficiency [27]. The distribution of participants according to each pattern differentiation for GERD will be described as the frequency and ratio using descriptive analysis.

### Safety and adverse event outcomes

#### Safety

Safety will be assessed using adverse reaction reports and clinical laboratory tests. Liver function tests include AST, ALT, alkaline phosphatase, total bilirubin, and γ-glutamyl transpeptidase levels, and renal function tests include blood urea nitrogen and creatinine levels. Women of childbearing age will be tested for pregnancy. This study will not include collection of any additional biological samples. Genetic or molecular analyses will not be performed.

#### Adverse events

An adverse event (AE) is an undesirable and unintended sign, symptom, or disease that does not necessarily have a cause-and-effect relationship with the intervention evaluated in a clinical trial. We will continuously monitor subjects for AEs and make all related decisions on the basis of both objective and subjective signs, as well as blood test results. All AEs that occur throughout the clinical trial will be recorded. The decision criteria for AE are as follows:

1. Grade 1 (mild): A grade 1 AE does not decrease the participant’s ability to perform normal activities of daily living (function) and only minimally brings discomfort to the participants and is easy to bear.
2. Grade 2 (moderate): A grade 2 AE causes discomfort that significantly decreases the participant’s ability to perform normal activities of daily living (function).
3. Grade 3 (severe or medically significant): A grade 3 AE renders the participant unable to perform activities of daily living.
4. Grade 4: life-threatening consequences
5. Grade 5: death related to AE

SAEs refer to any of the following AEs occurring in a participant during the clinical trial:

1. Death or danger to life
2. Hospitalization or extension of hospital stay due to an adverse event
3. Permanent or significant failure or degradation of function
4. Development of fetal malformations or abnormalities
5. Other medically important situations

The investigator rapidly reports all SAEs to the sponsor (usually within 24 h) in order to ensure patient safety at clinical trial and meet the MFDS guideline for reporting. The investigator also reports SAEs to the institutional review board.

### Data collection, management, and monitoring

Data and instrumental measurements will be collected from all subjects at every visit using a paper-based case report form. Data entry and management will be completed by an independent data administrator to ensure data accuracy. Only the principal investigator and subinvestigators delegated by the principal investigator can access the data. The final dataset is available only to the principal investigator and the independent statistician. All procedures will comply with the confidentiality standards for medical data. All documents related to the conduct of clinical trials will be retained by the principal investigator or subinvestigator. The participants’ information will be maintained in storage for a period of 3 years after study completion. Important protocol modifications during this study will be communicated to the institutional review board, trial registry, investigators, trial participants, and the journal of publication. An independent monitoring supervisor affiliated with the K-CTC will be assigned to contact and visit the researchers regularly and thus supervise the trial process.

The study is monitored by K-CTC employees. The monitoring is done to protect the participants’ rights and welfare; to prove whether reported data related to clinical trials are accurate, complete, and able to be validated when contrasted against evidence documents; and to check whether a proposal for an approved clinical trial and standard management and protocols of clinical trials are in compliance with regulations. The monitoring of meetings is performed two or three times annually. Besides, independent monitoring is conducted by the Korea Institute of Oriental Medicine, a sponsor of our study, in the first phase of patient recruitment and during the midphase of the study. This study is preliminary research conducted in a single institution in which the steering committee and data management team have not been organized. Management and analysis of data will be performed solely by an independent expert statistician.

### Sample size

This is a pilot study examining the feasibility of conducting a large-scale randomized clinical trial of OJS plus SMS for treating chronic cough in patients with GERD. A pilot study for planning a larger study and estimating its effect size requires an adequate small sample size. There is no accurate way to calculate a sample size for a pilot study; hence, we have adopted a study by Julious [28], and based on that, we have set the sample size at 24–12 in each group. Considering the rate of dropouts, a total of 30 was decided. On the basis of this pilot study, we plan to obtain an accurate estimation of the effect size and to conduct a larger-scale study.

### Statistical analysis

Statistical analysis will be performed using the SPSS statistical software package (version 18.0; SPSS, Inc., Chicago, IL, USA), and the level of significance will be established at α = 0.05. An independent professional statistician who is blinded to allocation will carry out the data analysis. Intention-to-treat (ITT) analysis will be used as the main analysis, and subordinately we will perform per-protocol (PP) analysis. The ITT population will include all participants who have been treated with at least one dose of the study drug and who record and keep a minimum of 1 day’s cough diary. The PP population will include all the participants of the study who have taken more than 80% of either the allocated investigational drug or the placebo drug and have returned their self-reported cough diary, which is the primary assessment variable, with a minimum of 34 days’ worth of assessment (more than 80% of the total 42 days of data evaluation). The cough diary will be used to compare the cough symptom scores at 6 weeks (visit 4). If the normality test is satisfied, the independent *t* test will be used; otherwise, the Mann-Whitney *U* test will be used. However, analysis of covariance will be performed if significant differences in the baseline cough diary values are found between the groups. Intergroup comparisons of cough VAS will be evaluated at weeks 2, 4, and 6. Mean differences in the LCQ-K, GSRS, and HARQ scores will be evaluated at baseline and week 6. Missing values will be replaced by the last observed value of each subject according to the last observation carried forward method. Interim analysis will not be performed for this study.

### Post-trial care

This clinical trial will adopt clinical trial insurance; if serious harm occurs following the clinical trial, the affected participants will receive appropriate coverage.

### Dissemination policy

We will disseminate the results of this clinical trial widely through conference presentations and publications in relevant journals. The data of this clinical trial will not be shared.

## Discussion

Approximately 10–59% of cases of chronic cough can be attributed to GERD [29]. To the best of our knowledge, this is the first clinical trial to explore the efficacy and safety of herbal medicines for reflux-related chronic cough, including patients without GI symptoms. Combination treatment such as OJS plus SMS is common in the clinical field for chronic cough due to GERD, but its scientific basis is not yet proven. Therefore, we aim to investigate the feasibility of OJS plus SMS for patients with chronic cough due to GERD. This will be a randomized, placebo-controlled, double-blind, parallel-arm, single-center clinical trial. This clinical trial was designed according to the Consolidated Standards of Reporting Trials guidelines [30]. Validated evaluation tools will be used to assess the severity and frequency of cough and to assess the effects of GERD and chronic cough on the patients’ quality of life. Pattern identification is used to reflect the clinical field. Using abdominal diagnostic methods, we will evaluate the symptoms (e.g., epigastric fullness) related to GI diseases that cause gastroesophageal reflux.

This study is a pilot study that will provide information about feasibility, the duration of patient recruitment, the dosing period, and whether there are problems that were not considered when planning a protocol, and so forth. In the resulting analysis, the primary outcome will be the effectiveness of the treatment on the cough symptom, and secondary outcomes will be the identification of reflux symptoms and to determine the relationship between the presence of the symptoms and the effect of the drug. In addition, we will try to identify the responder group, which is particularly responsive to the effect of the drugs, using pattern identification tools and abdominal diagnostic methods.

However, there are some limitations. First, there is no definitive way to diagnose GERD-induced cough with good levels of sensitivity and specificity [31]. Gastric endoscopy has a sensitivity of less than 20% among patients with reflux-associated chronic cough [32]. Twenty-four-hour esophageal pH monitoring or impedance/pH-metry can detect abnormal reflux, but these procedures are invasive and limited with respect to providing clear causal evidence [33]. Therefore, we are following the ACCP guideline to diagnose patients with chronic cough with GERD in accordance with this clinical profile [12]. It has been reported that 91% of patients diagnosed with this clinical profile were patients who responded to the antireflux treatment, despite the absence of GI symptoms [12]. We diagnosed GERD-induced chronic cough by conducting tests to eliminate other lung diseases, UACS, CVA, and eosinophilic bronchitis as the main causes of chronic cough in patients diagnosed with GERD. Therefore, we excluded smokers, subjects receiving angiotensin-converting enzyme inhibitor therapy, and those with abnormal thoracic radiography findings. Subsequently, we checked for other causes of chronic cough based on the ACCP guidelines and the Korean guidelines for chronic cough [34]. UACS, CVA, and eosinophilic bronchitis were checked for and excluded by paranasal sinus x-ray and nasal endoscopy, by PFT with bronchodilator test or methacholine bronchial challenge test, and by FeNO test, respectively. Second, the sample size was small and obtained within a single center, which is a characteristic of pilot studies. This study is being conducted to determine the sample size for validating large-scale studies with a pilot study and to assess the feasibility of the research. Based on the results of this study, large-scale studies will be planned with larger sample sizes.

We hope that this research will provide a scientific basis for the combination of herbal medicines, as well as identify the group that is responsive to OJS plus SMS. In the future, we expect to conduct a well-designed clinical trial that would clearly provide scientific evidence based on the results of this pilot study to confirm the efficacy and safety of OJS plus SMS for the treatment of GERD-induced chronic cough.

### Trial status

The study is currently in the process of recruiting participants. Recruitment of participants commenced on January 4, 2019, and will be completed by August 2020. The protocol version is KHMC-CCOS-P01, dated June 22, 2018.

## Supplementary information

**Additional file 1.** Informed consent form.

**Additional file 2.** SPIRIT 2013 checklist: recommended items to address in a clinical trial protocol and related documents

## Data Availability

The data of this clinical trial will not be shared.

## Abbreviations

ACCP: American College of Chest Physicians
AE: Adverse event
ALT: Alanine aminotransferase
AST: Aspartate aminotransferase
CVA: Cough variant asthma
FeNO: Fractional exhaled nitric oxide
GERD: Gastroesophageal reflux
GI: Gastrointestinal
GSRS: Gastrointestinal Symptom Rating Scale
HARQ: Hull Airway Reflux (hypersensitivity) Questionnaire
ITT: Intention to treat
K-CTC: Korean medical clinical trial center
KM: Korean medicine
LCQ: Leicester Cough Questionnaire
LCQ-K: Leicester Cough Questionnaire (Korean version)
MFDS: Ministry of Food and Drug Safety of Korea
OJS: *Ojeok-san*
PFT: Pulmonary function test
PICCQ: Pattern Identification for Chronic Cough Questionnaire
PP: Per protocol
PPI: Proton pump inhibitor
SAE: Serious adverse event
SMS: *Saengmaek-san*
UACS: Upper airway cough syndrome
VAS: Visual analogue scale

## References

[1] Pratter MR, Brightling CE, Boulet LP, Irwin RS (2006). An empiric integrative approach to the management of cough: ACCP evidence-based clinical practice guidelines. Chest..

[2] Morice AH, Fontana GA, Belvisi MG, Birring SS, Chung KF, Dicpinigaitis PV (2007). ERS guidelines on the assessment of cough. Eur Respir J.

[3] Kastelik JA, Aziz I, Ojoo JC, Thompson RH, Redington AE, Morice AH (2005). Investigation and management of chronic cough using a probability-based algorithm. Eur Respir J.

[4] Irwin RS, Baumann MH, Bolser DC, Boulet LP, Braman SS, Brightling CE (2006). Diagnosis and management of cough executive summary: ACCP evidence-based clinical practice guidelines. Chest..

[5] Mello CJ, Irwin RS, Curley FJ (1996). Predictive values of the character, timing, and complications of chronic cough in diagnosing its cause. Arch Intern Med.

[6] Poe RH, Kallay MC (2003). Chronic cough and gastroesophageal reflux disease: experience with specific therapy for diagnosis and treatment. Chest..

[7] Irwin RS, French CL, Curley FJ, Zawacki JK, Bennett FM (1993). Chronic cough due to gastroesophageal reflux: clinical, diagnostic, and pathogenetic aspects. Chest..

[8] Ing AJ, Ngu MC, Breslin AB (1994). Pathogenesis of chronic persistent cough associated with gastroesophageal reflux. Am J Respir Crit Care Med..

[9] Palombini BC, Villanova CA, Araujo E, Gastal OL, Alt DC, Stolz DP (1999). A pathogenic triad in chronic cough: asthma, postnasal drip syndrome, and gastroesophageal reflux disease. Chest..

[10] Irwin RS (2006). Chronic cough due to gastroesophageal reflux disease: ACCP evidence-based clinical practice guidelines. Chest..

[11] Laukka MA, Cameron AJ, Schei AJ (1994). Gastroesophageal reflux and chronic cough: which comes first?. J Clin Gastroenterol..

[12] Kahrilas PJ, Altman KW, Chang AB, Field SK, Harding SM, Lane AP (2016). Chronic cough due to gastroesophageal reflux in adults: CHEST guideline and expert panel report. Chest..

[13] Shaheen NJ, Crockett SD, Bright SD, Madanick RD, Buckmire R, Couch M (2011). Randomised clinical trial: high-dose acid suppression for chronic cough – a double-blind, placebo-controlled study. Aliment Pharmacol Ther..

[14] Lee SD, Kim EJ, Jung CY, Shin KM, Jang MK, Yoon EH (2010). Selection of adequate indicators for the development of a questionnaire to evaluate the effects of Ojeok-san [in Korean]. J Korean Med [Taehan Hanŭi Hakhoe chi]..

[15] Shin IS, Lee MY, Jeon WY, Kim JC, Shin HK (2013). Ojeok-san, a traditional Korean herbal medicine attenuates airway inflammation and pulmonary fibrosis induced by repeated ovalbumin challenge. J Ethnopharmacol.

[16] Ko KM (2002). Shengmai san.

[17] Wang L, Ju LT, Sai MT, Yang WJ (2011). Shengmai san for the treatment of connective tissue disease with pulmonary interstitial fibrosis [in Chinese]. China Mod Doctor [Zhongguo Xian Dai Yi Sheng].

[18] Yang AW, Lv HS, Zhang JL, Lin ZM, Liang RP (2016). Effect of *Shengmaisan* on chemotherapy side effects and symptoms in patients with advanced lung cancer [in Chinese]. Mod J Integr Trad Chin West Med [Xian Dai Zhong Xi Yi Jie He Za Zhi]..

[19] Kim BJ (2013). Shengmaisan regulates pacemaker potentials in interstitial cells of Cajal in mice. J Pharmacopuncture.

[20] Lee JH, Bhang YH, Kim JH, Do HY, Kim KI, Jung HJ (2017). Case study of three gastroesophageal reflux-induced chronic cough patients treated with Ojeok-san plus Saengmaek-san. J Int Korean Med..

[21] Ours TM, Kavuru MS, Schilz RJ, Richter JE (1999). A prospective evaluation of esophageal testing and a double-blind, randomized study of omeprazole in a diagnostic and therapeutic algorithm for chronic cough. Am J Gastroenterol.

[22] Birring SS, Prudon B, Carr AJ, Singh SJ, Morgan MD, Pavord ID (2003). Development of a symptom specific health status measure for patients with chronic cough: Leicester Cough Questionnaire (LCQ). Thorax..

[23] Han JM, Jung IC, Kang W, Kim SS, Yeo Y, Park YC (2014). Reliability and validity of Leicester Cough Questionnaire Korean version. Chron Respir Dis.

[24] Kulich KR, Madisch A, Pacini F, Pique JM, Regula J, Van Rensburg CJ (2008). Reliability and validity of the Gastrointestinal Symptom Rating Scale (GSRS) and Quality of Life in Reflux and Dyspepsia (QOLRAD) questionnaire in dyspepsia: a six-country study. Health Qual Life Outcomes.

[25] Morice AH, Faruqi S, Wright CE, Thompson R, Bland JM (2011). Cough hypersensitivity syndrome: a distinct clinical entity. Lung..

[26] Kim KI, Shin S, Lee NR, Lee BJ, Jung HJ, Jung SK (2015). Preliminary study for development of pattern identification tool of chronic cough. J Int Korean Med..

[27] Han GJ, Leem JT, Lee NR, Kim JS, Park JW, Lee JH (2015). Development of a standard tool for pattern identification of gastroesophageal reflux disease (GERD) [in Korean]. Korean J Intern Med [Taehan Naekwa Hakhoe Chapchi].

[28] Julious SA (2005). Sample size of 12 per group rule of thumb for a pilot study. Pharm Stat J Appl Stat Pharm Ind.

[29] Chang AB, Lasserson TJ, Gaffney J, Connor FL, Garske LA (2011). Gastro-oesophageal reflux treatment for prolonged non-specific cough in children and adults. Cochrane Database Syst Rev.

[30] Gagnier JJ, Boon H, Rochon P, Moher D, Barnes J, Bombardier C (2006). Recommendations for reporting randomized controlled trials of herbal interventions: explanation and elaboration. J Clin Epidemiol.

[31] Saritas Yuksel E, Vaezi MF (2012). Extraesophageal manifestations of gastroesophageal reflux disease: cough, asthma, laryngitis, chest pain. Swiss Med Wkly.

[32] Baldi F, Cappiello R, Cavoli C, Ghersi S, Torresan F, Roda E (2006). Proton pump inhibitor treatment of patients with gastroesophageal reflux-related chronic cough: a comparison between two different daily doses of lansoprazole. World J Gastroenterol.

[33] Nam SJ, Park SC, Lee SJ (2016). Extraesophageal manifestations of gastroesophageal reflux disease [in Korean]. Korean J Med.

[34] Song DJ, Song WJ, Kwon JW, Kim GW, Kim M, Kim MY (2018). KAAACI evidence-based clinical practice guidelines for chronic cough in adults and children in Korea. Allergy Asthma Immunol Res.

