# Supplementary material for: Efficacy and safety of Ojeok-san plus Saengmaek-san for gastroesophageal reflux-induced chronic cough: protocol for a pilot, randomized, double-blind, placebo-controlled trial
Source: Trials. 2020 Jan 29;21:118. doi: 10.1186/s13063-019-4030-z (PMC6990619; doi:10.1186/s13063-019-4030-z)
Supplement: Supplementary file 1 — Additional file 1. Informed consent form. [file 13063_2019_4030_MOESM1_ESM.docx]

**Informed Consent form**

| *** | **Please read the following information carefully and listen to the researcher for further explanations.**  **If you voluntarily agree, please check the boxes (☑).** | *** |
| --- | --- | --- |

1. I have read the description of this study and have discussed it with the principal investigator. □
2. I have been adequately informed about the risks and benefits of the study and I have received answers that satisfied my questions. □
3. I have been fully informed by the investigator about the benefits and risks associated with this study and I have been informed that I can always ask the investigator for a full explanation. □
4. I voluntarily agree to participate in this study. □
5. I understand that I may discontinue my participation in this study at any time without penalty or loss of benefits □
6. I consent that my information obtained from the study can be collected and processed by the researcher to the extent permitted by current laws and regulations of the Institutional Review Board □

**<Personal Information Collection/Usage History>**

| **Categories of personal information** | **Purpose of Collection** | **Retention Period** |
| --- | --- | --- |
| **Date of Birth** | **Research** | **24 months**  **(Until May 2020)** |

**※You have the right to refuse to provide your personal information.**

**☞ Do you agree to the collection and use of personal information as above? (□ yes, □ no )**

**<Sensitive Information Collection/Usage History>**

| **Categories of sensitive Information** | **Purpose of collection** | **Retention period** |
| --- | --- | --- |
| **Health (current and past medical history, drug utilization, etc.)** | **Research** | **24 months**  **(Until May 2020)** |

**※You have the right to refuse to provide your sensitive information**

**☞ Do you agree to the processing of sensitive information as above? ( □ yes, □ no)**

7. I agree that my medical records may be accessed, to the extent that my personal information is protected, in cases of conducting tests and managing results by the researchers and authorized representatives and verifying the quality of my personal information by monitoring personnel, inspectors, institutional bioethics committee, the Minister of Health and Welfare and the Director of Food and Drug Safety in accordance with the relevant laws and reregulation. I also agree that the consent signed by me and my representative permits access to such information and materials. □

8. My signature indicates that I have received a copy of this manual and consent form and I will keep my copy until the end of this study **□**

**I fully understand the contents of the consent form and I hereby consent to the agreements by signing below.**

| Participants | Name: | Signature: | Date: / / | |
| --- | --- | --- | --- | --- |
| Legal representative(when necessary) | Name: | Signature: | Date: / / | |
|  | *Relation to the subject: (Parents ‧ Spouse ‧ Guardian) | | | |
|  | *Reason: | | | |
| Observer  (when necessary) | Name: | Signature: | | Date: / / |
| Principal Investigator | Name: | Signature: | | Date: / / |
